# Distinguishing NASH Histological Severity Using a Multiplatform Metabolomics Approach

**DOI:** 10.3390/metabo10040168

**Published:** 2020-04-24

**Authors:** George N. Ioannou, G. A. Nagana Gowda, Danijel Djukovic, Daniel Raftery

**Affiliations:** 1 Division of Gastroenterology, Veterans Affairs Puget Sound Healthcare System and University of Washington, Seattle, WA 98108, USA; 2 Department of Medicine, Veterans Affairs Puget Sound Healthcare System and University of Washington, Seattle, WA 98108, USA; 3 Research and Development, Veterans Affairs Puget Sound Healthcare System, Seattle, WA 98108, USA; 4 Northwest Metabolomics Research Center, Anesthesiology and Pain Medicine, University of Washington, Seattle, WA 98109, USA; ngowda@uw.edu (G.A.N.G.); djukovic@uw.edu (D.D.); 5 Department of Chemistry, University of Washington, Seattle, WA 98195, USA; 6 Fred Hutchinson Cancer Research Center, Seattle, WA 98109, USA

**Keywords:** nonalcoholic fatty liver, nonalcoholic steatohepatitis, liquid chromatography-mass spectrometry, nuclear magnetic resonance spectroscopy, metabolic pathway

## Abstract

Nonalcoholic fatty liver disease (NAFLD) is categorized based on histological severity into nonalcoholic fatty liver (NAFL) or nonalcoholic steatohepatitis (NASH). We used a multiplatform metabolomics approach to identify metabolite markers and metabolic pathways that distinguish NAFL from early NASH and advanced NASH. We analyzed fasting serum samples from 57 prospectively-recruited patients with histologically-proven NAFLD, including 12 with NAFL, 31 with early NASH and 14 with advanced NASH. Metabolite profiling was performed using a combination of liquid chromatography-mass spectrometry (LC-MS) and nuclear magnetic resonance (NMR) spectroscopy analyzed with multivariate statistical and pathway analysis tools. We targeted 237 metabolites of which 158 were quantified. Multivariate analysis uncovered metabolite profile clusters for patients with NAFL, early NASH, and advanced NASH. Also, multiple individual metabolites were associated with histological severity, most notably spermidine which was more than 2-fold lower in advanced fibrosis vs. early fibrosis, in advanced NASH vs. NAFL and in advanced NASH vs. early NASH, suggesting that spermidine exercises a protective effect against development of fibrosing NASH. Furthermore, the results also showed metabolic pathway perturbations between early-NASH and advanced-NASH. In conclusion, using a combination of two reliable analytical platforms (LC-MS and NMR spectroscopy) we identified individual metabolites, metabolite clusters and metabolic pathways that were significantly different between NAFL, early-NASH, and advanced-NASH. These differences provide mechanistic insights as well as potentially important metabolic biomarker candidates that may noninvasively distinguish patients with NAFL, early-NASH, and advanced-NASH. The associations of spermidine levels with less advanced histology merit further assessment of the potential protective effects of spermidine in NAFLD.

## 1. Introduction

Nonalcoholic fatty liver disease (NAFLD) is strongly associated with the metabolic syndrome and each of its components [1,2]. In fact, NAFLD is regarded as the hepatic manifestation of the metabolic syndrome [1]. By definition, all patients with NAFLD have excessive lipid deposition within lipid droplets in hepatocytes, a condition known as hepatic steatosis. However, patients with NAFLD can exhibit a very broad spectrum of hepatic inflammation and fibrosis ranging from almost none (a condition known as simple steatosis or “nonalcoholic fatty liver” (NAFL)) to variable degrees of inflammation and fibrosis (known as “nonalcoholic steatohepatitis” or “NASH”) to established cirrhosis [3,4]. Patients with NAFL have a very low probability of progression to cirrhosis or hepatocellular carcinoma (HCC). On the other hand, patients with NASH have significantly higher probability of progression [4,5]. Furthermore, among the histological features that distinguish NASH, hepatic fibrosis is emerging as the feature most strongly associated with adverse liver-related outcomes and even all-cause mortality, rather than inflammation or the presence of balloon hepatocytes [6,7]. Therefore, there is a pressing clinical need to understand the factors that lead to the development of fibrosing NASH, rather than NAFL and to identify non-invasive tests that can distinguish the presence of NASH versus simple steatosis. Given the strong link between metabolic factors and NAFLD, we hypothesized that serum metabolite profiling using metabolomics could identify serum metabolites that distinguish NAFL from NASH.

The growing field of metabolomics describes the study of concentrations and fluxes of low molecular weight metabolites (molecular weight <1000 Da) present in biofluids or tissue that provide detailed information for understanding biological phenotypes, deciphering mechanisms, and identifying biomarkers or drug targets for a variety of conditions [8,9,10]. Utilization of two powerful analytical platforms, liquid chromatography-mass spectrometry (LC-MS) [11] and nuclear magnetic resonance (NMR) [12] spectroscopy, leads to the quantitative analysis of hundreds of small molecules on a relatively routine basis. In the area of liver diseases, to date, several cross-sectional studies have demonstrated the potential for serum or plasma-derived metabolite biomarkers to distinguish different liver diseases and hepatocellular carcinoma (HCC) [13,14,15,16,17,18,19]. Metabolomics studies in this area have focused on both early biomarker detection as well as identification of altered metabolic pathways [20]. It has been shown that metabolite-based biomarkers can distinguish HCC from cirrhosis better than the conventional marker, alpha-fetoprotein (AFP) [14,21]. However, metabolomic investigations focused on distinguishing high risk NASH from NAFL are scarce.

In the current study, metabolite profiling of serum samples employing both MS and NMR spectroscopy methods, in combination with multivariate statistical methods, was performed to identify serum metabolites or metabolite profiles that distinguish simple steatosis from NASH, or even early NASH from advanced NASH. Our results show promise for developing metabolite profiles to stage patients as well as providing insights on the pathogenesis and progression of NAFLD.

## 2. Results

### 2.1. Patient Characteristics

Among 57 patients with biopsy-proven NAFLD included in this study, the mean age was 51 years and the majority were white and male—consistent with recruitment from a VA hospital (Table 1). As expected in patients with NAFLD, diabetes was very common, mean BMI was in the range of obesity and mean serum AST and ALT were mildly elevated (Table 1).

By study design, patients with early-NASH and advanced-NASH had progressively increasing NAS, ballooning degeneration, inflammation, steatosis, and fibrosis scores compared to patients with simple steatosis. Patients with early-NASH had a mean NAS score of 3.3 ± 0.7 and fibrosis stage of 0.8 ± 0.6. Patients with advanced-NASH had a mean NAS score of 5.7 ± 0.6 and fibrosis stage of 2.0 ± 0.9.

### 2.2. Serum Metabolite Profiles Cluster According to NAFL, Early-NASH and Advanced-NASH

**Figure 1:**
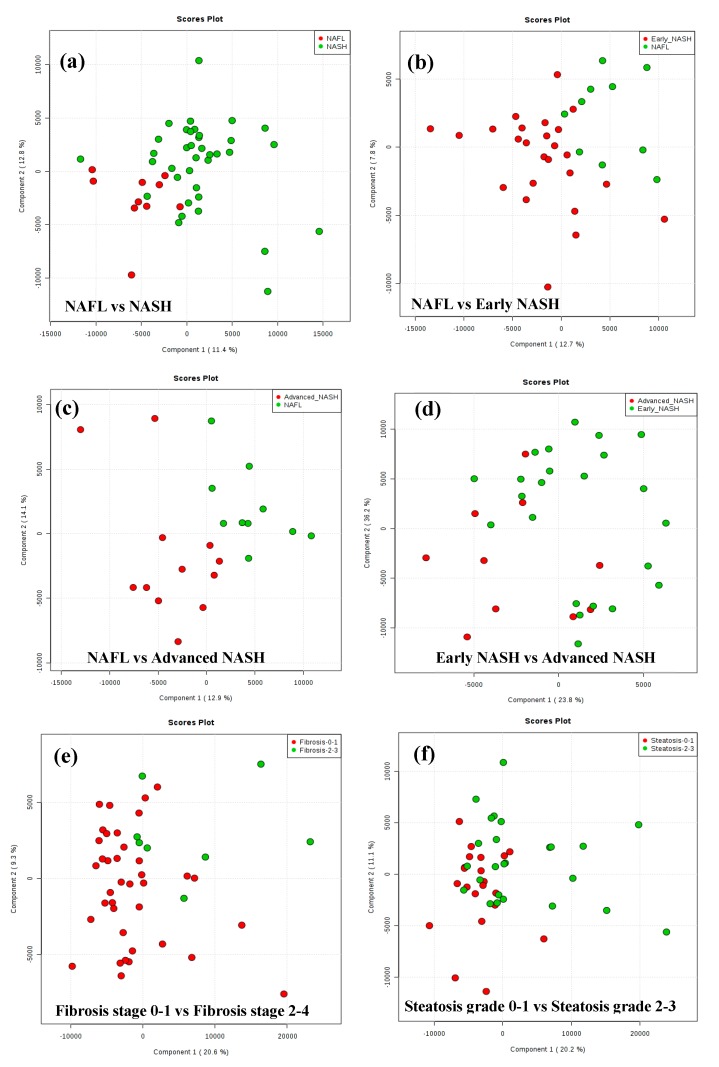
The results of PLS-DA analysis of metabolite levels derived from MS are shown as score plots. Each red or green point in the score plot represents one patient. In these plots, points (known as scores) for patients that exhibit similar metabolite profiles will appear closer to each other and vice versa. The score plots show good clustering and separation of the patients with (**a**) nonalcoholic fatty liver (NAFL) vs. NASH; (**b**) NAFL vs. early NASH; (**c**) NAFL vs. advanced NASH; (**d**) Early NASH vs. advanced NASH; (**e**) Fibrosis stage 0–1 vs. Fibrosis stage 2–4; and (**f**) Steatosis grade 0–1 vs. Steatosis grade 2–3.

Serum metabolite profiling using LC-MS and NMR techniques targeted 237 metabolites. Of these, 158 metabolites were quantified, 106 metabolites by MS (Table S1) and 52 metabolites by NMR (Table S2). Partial least squares-discriminant analysis (PLS-DA) results are shown as score plots in Figure 1. Each circle or point in the score plot represents one patient. The points (also known as scores) for patients that exhibit similar metabolite profiles in serum appear closer to each other and vice versa. Ideally, the scores for patients with NAFL, early NASH and advanced NASH will form separate and distinct clusters owing to the potential differences in metabolic profiles between the groups. In our study, multivariate analysis showed clustering of the serum metabolite profiles of patients depending on the histological severity of NAFLD (NAFL vs. NASH and early-NASH, vs. advanced-NASH) as shown in Figure 1a–c. In particular, the metabolite profiles of NAFL and NASH showed a distinct clustering with minimal overlap of the scores for patients (Figure 1a); the dispersion of the scores was greater for NASH compared to NAFL. However, results for the analysis of the data after dividing NASH patients into early NASH and advanced NASH showed significant improvement in the separation; both early NASH vs. NAFL as well as advanced NASH vs. NAFL exhibited distinct clusters (Figure 1b,c).

Multivariate statistical results also reflected the metabolic differences between early NASH and advanced NASH; a comparison between the two groups showed separate clusters, although there was some degree of overlap (Figure 1d). In addition, analysis performed after subdividing the patients according to fibrosis stage (0–1 vs. 2–4) or steatosis grade (0–1 vs. 2–3) also showed clusters, although the two clusters were again partially overlapped (Figure 1e,f).

### 2.3. Individual Metabolites were Associated with Histological Severity in NAFLD

Univariate analysis identified 11 metabolites that were significantly different between NAFL and NASH with fold changes ranging from 0.57 to 3.13 (Table 2). Nine metabolites were significantly different between NAFL and early-NASH (with fold changes from 0.83 to 1.19) and ten were significantly different between NAFL and advanced-NASH (with FC from 0.33 to 2.38). Six metabolites were significantly different between early-NASH and advanced-NASH with FC from 0.49 to 1.33; further, the metabolites that distinguished early-NASH from advanced-NASH were largely different from the ones that distinguished NAFL from early-NASH (Table 2). In addition, we identified eight metabolites that were significantly different between fibrosis stages 0–1 and fibrosis stages 2–4 and four significantly different between steatosis grades 0–1 and steatosis grades 2–3. (Table 3). The most notable example was spermidine, which was more than 2-fold decreased in advanced fibrosis vs. early fibrosis, in advanced NASH vs. NAFL, and in advanced NASH vs. early NASH, suggesting that spermidine exercises a protective effect against development of fibrosing NASH.

### 2.4. Metabolic Pathways and Metabolite Enrichment Distinguished NAFL, Early-NASH, Advanced-NASH

Metabolic pathway enrichment analysis was performed using the Metaboanalyst software platform [22,23,24]. The results are shown in Figure S1, where each pathway is shown as a circle. The color of the circle indicates the significance of the pathway with red being the most significant (*p* < 0.05) and the size of the circle indicates the impact of the pathway. Pathway analysis made based on 106 MS derived metabolite levels showed a total of 56 matched pathways and the analysis based on 52 NMR derived metabolites showed 48 matched metabolic pathways. A number of these pathways were significantly altered (*p* < 0.05) between different groups of patients (Table S3). All comparisons except NAFL vs. advanced NASH showed significant differences in at least one pathway. In particular, the differences between early-NASH and advanced-NASH, and between steatosis grade 0,1 and steatosis grade 2,3 were more significant than between the other groups; the former exhibited the most number of altered pathways and the latter exhibited the most number of pathways that were highly significant (*p* ≤ 0.003) (Table S3, Figure S1).

**Figure 2:**
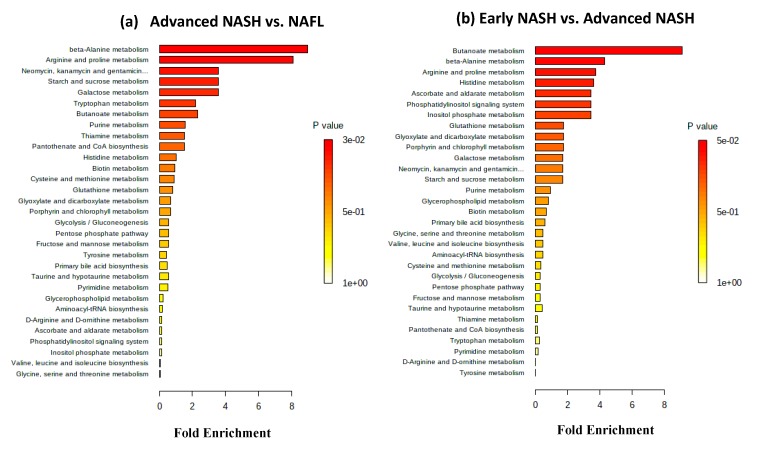
Biological patterns identified from metabolite set enrichment analysis. Metabolite data were derived from MS. The enrichment analysis combines functionally related metabolites to discern consistent changes among the related metabolites. The color and the bar length indicate *p* value and the fold enrichment, respectively. (**a**) In the advanced NASH vs. NAFL comparison, the analysis identified 31 sets, of which two sets that correspond to beta-alanine metabolism and arginine and proline metabolism exhibited high significance; the *p* value adjusted using false discovery rate for both sets was 0.02. (**b**) In the early NASH vs. advanced NASH comparison, 31 sets were also identified, of which one set that corresponds to butanoate metabolism exhibited high significance; its *p* value adjusted using false discovery rate was 0.05.

Separately, metabolite set enrichment analysis was performed using quantitative metabolite data. Both advanced NASH vs. NAFL and early NASH vs. advanced NASH identified 31 sets of metabolites (Figure 2; Figure S2). For advanced NASH vs. NAFL, two sets that correspond to beta-alanine metabolism and arginine and proline metabolism exhibited high significance; the *p* value adjusted using FDR for both sets was 0.02 (Figure 2a). On the other hand, for early NASH vs. advanced NASH (Figure 2b), one set that corresponds to butanoate metabolism exhibited high significance; as shown in the figure, its *p* value adjusted using FDR was 0.05.

### 2.5. Association of the GSG-Index with Histological Severity

Neither the GSG-index nor its amino acids, individually, discriminated between NAFLD and NASH (data not shown). They also did not discriminate either between mild (stage 0, 1) and advanced fibrosis (stage 2–4) or between mild (grade 0, 1) and advanced steatosis (grade 2, 3). However, the alanine, aspartate, and glutamate metabolism pathway, which involves one of the amino acids of the GSG index was altered between early NASH and advanced NASH and between hepatic fibrosis stage 0–1 and Fibrosis stage 2–3 (Table S3). In addition, glycine, serine, and threonine metabolism, which involves glycine as one of the amino acids of the GSG index was altered between hepatic fibrosis stage 0–1 and Fibrosis stage 2–4.

### 2.6. Evaluation of the Confounding Effects of Gender and Diabetes on the Biomarkers of NASH/NAFL

Female patients were minimally differentiated in the multivariate statistical analysis results as shown in the Supplementary Figure S3a. Univariate analysis results showed that 17 metabolites were statistically different (*p* < 0.05) between male vs. female patients (Table S4). However, none of these metabolites was common to the biomarkers of NASH/NAFL disease patients (Table 2). Similarly, evaluation of metabolite signatures that differ between diabetes and non-diabetes patients showed minor differences as seen from the partial overlap of the two clusters in the Supplementary Figure S3b. Ten metabolites were statistically different between diabetes and non-diabetes patients (Table S5), of which 4 metabolites (isovaleric acid, oxypurinol, xanthine and sucrose) were common to those observed for NASH/NAFL disease (Table 2).

## 3. Discussion

There is great interest in identifying patients with NAFLD who are at risk of having, or progressing to, histologically advanced disease. Our goal was to identify serum metabolite profiles or metabolite pathways that distinguished NAFL, early-NASH and advanced-NASH using targeted LC-MS and quantitative NMR-based metabolomics approaches, combined with multivariate statistical methods. Serum samples obtained from patients after overnight fasting were used to avoid potential interferences from diet. For a global visualization of the data, multivariate statistical analysis was utilized and showed distinct clustering of patients, which provides evidence for the metabolic differences between NAFLD patients grouped by histological severity (Figure 1). In particular, the separation of the clusters between NAFL and NASH reveals a large difference in metabolism between these groups and the ability of the serum markers to identify the high risk NASH patients. We also found a significant difference between early NASH and advanced NASH, which indicates the potential to identify the patients at highest risk.

The results that many individual metabolite levels are significantly different (*p* < 0.05) between patients with NASH vs. NAFL not only substantiate the results of multivariate statistical analysis but also provide more specific information on altered cellular metabolism (Table 2). Among the many metabolites that were altered significantly, notable metabolic perturbations are the more than 2-fold reduction of spermidine and acetylglycine, and the more than 2-fold increase of oxypurinol, xanthine and bile acids (glycocholate and glycochenodeoxycholate) in advanced NASH compared to NAFL or early NASH (Table 2). Spermidine, which is found in mushrooms, aged cheese, soybeans, legumes and whole grains, has recently been shown to prevent liver fibrosis and hepatocellular carcinoma and to ameliorate fatty liver disease in mouse models [25]. However, we are not aware of other human studies suggesting a protective effect of spermidine against NASH. We found that levels of spermidine were more than two times lower consistently when comparing advanced fibrosis vs. early fibrosis, or advanced NASH vs. NAFL, or advanced NASH vs. early NASH. This suggests that spermidine might exercise a protective effect against development of fibrosing NASH and should be confirmed in future human studies.

A number of studies have shown the association of acetylglycine with liver disease; for example, its increased level was observed in liver tissue, serum as well as urine due to drug induced injury to the liver [26] or liver disease induced by alcohol [27,28]. Oxypurinol is an inhibitor of xanthine oxidase and is monitored to assess liver function [29]. The large changes observed for these metabolite levels in our study indicates their potential utility as distinguishing markers in clinical management of the NASH patients, as such large perturbations are more likely to hold upon independent validation.

Metabolic pathway analyses enabled global visualization of alterations in metabolism between the NAFLD patient groups. In particular, the results indicate that while the measured serum metabolites mapped to a large number of metabolic pathways, only six were significantly different between NASH and NAFL (Table S3). The most significant among the pathways is fatty acid biosynthesis. Our results indicate that the fatty acid synthesis pathway is associated with NAFLD, which is in accordance with previous findings that showed hepatic accumulation of triglycerides [30]. Taurine and hypotaurine metabolism is another pathway that distinguishes NASH from NAFL. It is well known that bile acid synthesis is one of the major functions of the liver and taurine conjugated bile acids are a major component in human bile [31]. Altered metabolism of taurine may indicate its association with altered bile acids synthesis in NASH. In accordance with the altered pathway, our univariate analysis results show that two primary bile acids, glycocholate and glycochenoxycholate differ significantly between advanced NASH and simple steatosis; both bile acids were higher in NASH by more than 2-fold (Table 2).

Metabolite set enrichment analysis enabled identification of biologically meaningful patterns for NAFLD patients. This analysis combines functionally related metabolites to discern consistent changes among the related metabolites. Although, patterns with high significance (*p* < 0.05) were identified for many groups of NAFLD patients, only two groups, advanced NASH vs. NAFL and early NASH vs. advanced NASH, showed patterns that exhibited high significance (*p* < 0.05) even after adjusting the *p* value using the false discovery rate (FDR) (Figure 2; Figure S2). Beta-alanine metabolism and arginine and proline metabolism were significantly altered for advanced NASH vs. NAFL (Figure 2a), whereas butanoate metabolism was significantly altered for early NASH vs. advanced NASH (Figure 2b). In terms of specific metabolites, spermidine contributed the most for advanced NASH vs. NAFL, which is in agreement with its down regulation in advanced NASH vs. NAFL (Table 2). On the other hand, 2-hydroxyglutrate contributed the most for early NASH vs. advanced NASH, which is also in accordance with its upregulation in advanced NASH vs. early NASH (Table 2).

We also found that metabolism is altered significantly depending on the degree of hepatic fibrosis or steatosis. These differences were significant and observed in the: (A) multivariate statistical analysis results, where clusters were clearly visible (Figure 1e,f); (B) univariate analysis results, where many metabolites are significantly different (Table 3); and (C) metabolic pathway analysis, where several pathways were significantly impacted (Figure S1d,e, Table S3). Overall, the results indicate that metabolic profiling is sensitive to differences in fibrosis and steatosis, the two most important histological features of NAFLD.

Numerous studies indicate serum amino acids levels, particularly, glutamate, glycine and serine, are altered in liver diseases [32,33,34,35,36,37]. More recently, the ratio of glutamate to the sum of serine and glycine, termed the GSG-index, was tested for its association with blood levels of BCCAs, ALT, AST, and GGT [38]. Amino acids of the GSG-index are critical for the synthesis of glutathione (GSH), which is the major antioxidant that controls oxidative stress in the liver [39]. The GSG-index was moderately correlated with ALT (r = 0.34 ± 0.13) and AST (r = 0.45 ± 0.12). However, in our study the GSG-index did not discriminate between NAFLD vs. NASH or between mild (stage 0–1) and more advanced (stage 2–4) liver fibrosis. In the prior study the GSG-index was shown to be significantly associated with stage 3–4 vs. stage 0–2 fibrosis (rather than stage 2–4 vs. 0–1 as we tested). Future, larger studies will need to further evaluate whether the GSG-index is a marker of histological severity in NAFLD.

Considering the lack of metabolomics studies that focus on identifying biomarkers that distinguish NASH from NAFLD, our results that demonstrate numerous altered metabolites and metabolic pathways between the two groups are potentially of high significance. In prior studies, urine and serum were used to distinguish different stages of NAFLD based on metabolomics [40]. Some of the findings, such as the higher levels of xanthine and tryptophan in NASH as well as the association of energy metabolism and amino acid metabolism with the pathological processes in NASH are in accordance with our findings (Table 2, Table S3). Another recent study using mice as well as patient samples identified several metabolites that distinguished simple steatosis from NASH [41]. Metabolic signatures including fatty acids, amino acids and bile acids that differentiate NAFL and NASH were detected, and these findings are somewhat in accordance with our results. A study of blood plasma, rather than serum, showed metabolic differences between controls vs. NAFLD as well as control vs. NASH; however, it could not identify a distinguishing metabolite profile between steatosis and NASH [32]. In that study, global LC/MS and GC-MS methods were used, while in our study the more quantitative targeted LC-MS and NMR platforms were used to analyze the determined pool of pre-identified metabolites, unambiguously. It is important to note that in the metabolomics field different analytical platforms have been used to investigate the same disease; depending on the sensitivity, selectivity, and reliability of metabolite identification, different analytical platforms enable access to different facets of the metabolome. In this study the combined use of the robust LC-MS and NMR methods for quantitative analysis of metabolites represents a broad based approach. In our study, apart from differentiating between NAFL and NASH, we have identified metabolites and metabolic pathways that differentiate various subtypes of NAFLD including early NASH and advanced NASH, as well as fibrosis stage and steatosis grade, and find many differentiating metabolites and pathways among the different histologies. Separately, analysis of the metabolite profiles showed that the confounding effects of demographics such as gender and diabetes are minimal in the biomarker identification for NAFL disease. To our knowledge, this is the first study to identify distinguishing metabolites and metabolic pathways for different liver histology associated with NAFL, combining targeted LC-MS and NMR based metabolomics methods.

Many other “non-metabolite”, serum-based biomarkers (e.g., type 4 collagen, M2BPGi) or biomarker panels (e.g., Fibrosis-4 score, Enhanced Liver Fibrosis score, Fibrotest/Fibrosure, Fibrospect) have been described that may distinguish advanced NASH, early NASH and NAFL [42]. Future studies should compare the performance characteristics of these tests with the metabolites that we described in distinguishing disease severity in NAFLD.

There are several limitations to this study, most notably the moderate sample size. Our results of novel metabolite (e.g., spermidine) and metabolite pathways associated with NASH severity will need to be confirmed in future studies. The small number of subjects does not allow us to make conclusions as to the separate effects of diabetes and insulin resistance, as insulin resistance was not measured in this study. Although two widely used metabolomics platforms, MS and NMR, were used to profile serum metabolites, the number of metabolites profiled is still small compared to the breadth of the serum metabolite profile. Moreover, no independent validation of the results was made.

In conclusion, a comprehensive metabolite profiling combining data from two analytical platforms, MS and NMR, provides new insights into the underlying metabolites and metabolic pathways that distinguish NAFLD patients based on histological severity. In particular, numerous features distinguished high risk NASH from low risk NAFL that potentially offers new avenues to identify high-risk NASH patients in the clinical setting. Of significance is that apart from identifying many altered metabolic pathways, our study enabled the identification of metabolites such as spermidine, acytylglycine, oxypurinol, and bile acids that varied by as much as 2-fold or higher between NAFL and NASH. These findings are particularly remarkable considering the tight regulation of many blood metabolites.

## 4. Materials and Methods

### 4.1. Study Population

All subjects gave their informed consent for inclusion before they participated in the study. The study was conducted in accordance with the Declaration of Helsinki, and the protocol was approved by the Institutional Review Board (IRB) at the Veterans Affairs Puget Sound Healthcare System (IRB protocol # 01010). Patients were identified from a prospective biorepository of chronic liver disease patients established at the Veterans Affairs Puget Sound Healthcare System, which recruited patients undergoing clinically indicated liver biopsy. We identified patients with NAFLD based on histological hepatic steatosis from liver biopsies in the absence of hepatitis C virus (negative serum HCV antibody and HCV RNA), hepatitis B virus (negative serum HBV surface antigen), excessive alcohol consumption (dedicated alcohol questionnaire administered on the day of liver biopsy), iron overload (hepatic stain and serum iron markers), or markers of autoimmune liver diseases. The liver biopsies of these patients with NAFLD were prospectively reviewed by a single hepatopathologist who scored the grade of steatosis (1–3), inflammation (0–3) and ballooning degeneration (0–2) and the stage of fibrosis (0–4) according to the system proposed by Kleiner et al. [43]. Based on these scores we divided the patients with NAFLD a priori according to their histological severity into the following groups, without any knowledge of their metabolomic profiles:

- NAFL (*n* = 12): Steatosis grade 1–3 with no or minimal inflammation (grade 0–1), no ballooning degeneration (grade 0) and no fibrosis (stage F0).
- Early-NASH (*n* = 31): No or mild fibrosis (stage F0-F1) and NAFLD activity score (NAS) 3–4, including steatosis grade ≥1, inflammation ≥1, and ballooning degeneration score ≥1. The NAS score is the sum of steatosis (0–3) plus inflammation (0–3) plus ballooning degeneration (0–2) grades and takes values ranging from 1–8 [43].
- Advanced-NASH (*n* = 14): Fibrosis score F1-F4 and NAS score 5–8, including ballooning degeneration score = 2.

The European Association for the Study of the Liver (EASL) defines “early” NASH as “no or mild fibrosis F0-F1” [44], which is consistent with the definition we chose above. However, there is no universally agreed definition of “advanced” NASH and we wanted to categorize NASH based on both fibrosis and NAS scores, hence we used the categories above.

In addition, we performed analyses based on subdividing patients by fibrosis stage (0–1 vs. 2–4) and by steatosis grade (0–1 vs. 2–3), given the fundamental importance of these histological features.

### 4.2. Serum Metabolite Profiling: LC-MS

A fasting serum specimen was prospectively collected from all patients between 7–9 am after an overnight fast just prior to liver biopsy and stored at −80 °C until the analysis.

Frozen serum samples were thawed at 4 ^o^C, after which protein precipitation and metabolite extraction was performed by adding 150 μL of methanol; the mixture was then vortexed for 2 min, stored at −20 °C for 20 min and centrifuged at 20,800× *g* for 10 min. The supernatant was collected into a new Eppendorf vial and dried using a Vacufuge Plus evaporator (Eppendorf, Hauppauge, NY, USA). The dried samples were stored at −20 °C and were reconstituted in 500 μL of 5 mM ammonium acetate in 40% water/60% acetonitrile + 0.2% acetic acid containing 5.13 μM tyrosine-^13^C_2_ and 22.5 μM sodium lactate-^13^C_3_. The two isotope-labeled internal standards were added to each sample to monitor the MS system performance. The samples were filtered through 0.45 μm PVDF filters (Phenomenex, Torrance, CA, USA) prior to LC−MS analysis. A pooled human serum extracted using the same procedure as described above was used as the quality-control (QC) sample and was analyzed once every 10 patient samples.

Targeted LC-MS analysis was performed using an AB Sciex QTrap 5500 mass spectrometer (AB Sciex, Toronto, ON, Canada) equipped with an electrospray ionization (ESI) source. The MS instrument was connected to an LC system composed of two Agilent 1260 binary pumps, an Agilent 1260 autosampler, and Agilent 1290 column compartment containing a column-switching valve (Agilent Technologies, Santa Clara, CA, USA). Two hydrophilic interaction chromatography (HILIC) columns (SeQuant ZIC-cHILIC columns; 150 × 2.1 mm, 3.0 μm particle size, Merck KGaA, Darmstadt, Germany) connected in parallel were used. One column was used for positive ionization and the other, for negative ionization. Each sample was injected twice, once for positive ionization mode (2 μL) and once for negative ionization mode (10 μL). Targeted data acquisition was performed in multiple-reaction-monitoring (MRM) mode. The mobile phase, the gradient conditions and the MS parameters used have been described previously in detail [45]. The extracted MRM peaks were integrated using MultiQuant 2.1 software (AB Sciex).

### 4.3. NMR Spectroscopy

NMR analyses were performed on a Bruker Avance III 800 MHz spectrometer equipped with a cryogenically cooled probe and Z-gradients suitable for inverse detection. The CPMG (Carr-Purcell-Meiboom-Gill) pulse sequence with water suppression using presaturation, was used with 9615 Hz spectral width, 32 k time domain points, 6 s relaxation delay and 256 transients for each sample. Fourier transformation was performed using a spectral size of 32 k data points after multiplying the raw data by an exponential window function with line broadening of 0.5 Hz. Chemical shifts were referenced to the internal TSP signal for ^1^H 1D spectra. Bruker Topspin software (version 3.0 or 3.1) was used for NMR data acquisition, processing and analyses. Peak assignments relied on established literature values [46]. Bruker AMIX software was used to quantitate metabolites.

### 4.4. Statistical, Enrichment, and Pathway Analyses

Relative peak integrals or (where available) absolute concentrations of metabolites obtained from MS and NMR were used for data analysis. The MS and NMR data were subjected to univariate (Student’s *t*-test) analysis, multivariate analysis, principle component analysis (PCA), partial least squares discriminant analysis (PLS-DA), statistical correlations, and metabolic pathway analysis. Prior to statistical analysis the MS and NMR data were normalized using Pareto scaling, where each variable (concentration for each metabolite) was mean-centered and divided by the square root of standard deviation of each variable. Metabolites with *p* < 0.05 were considered statistically significant. Pathway enrichment analysis was performed to identify pathways significantly associated with the different groups of NAFLD patients. For multivariate analysis, statistical correlations, enrichment analysis and pathway analysis, the Metaboanalyst software package version 4.0 [22,23,24] was used. We have also evaluated whether the confounding factors such as gender and diabetes affected the biomarkers of NAFLD.

### 4.5. Evaluation of the GSG-Index

The GSG-index, a newly developed parameter associated with NAFLD, refers to the ratio of glutamate to sum of serine and glycine [glutamate/(serine + glycine)] [38]. Amino acids of the GSG-index, glutamate, serine, and glycine, are critical for the synthesis of glutathione (GSH), which is the major antioxidant that controls oxidative stress in the liver. Several amino acids including those associated with the GSG-index, branched chain amino acids (BCCAs) and aromatic amino acids (AAAs) were shown to be altered in NAFLD [38]. Hence, we evaluated the associations of the GSG-index with histological severity of NAFLD.

## Figures and Tables

**Table 1:** Demographic and clinical characteristics of study participants divided according to histological severity of nonalcoholic fatty liver disease (NAFLD) (simple steatosis, early-nonalcoholic steatohepatitis (NASH), advanced-NASH).

Parameter	Simple Steatosis *n* = 12	Early NASH *n* = 31	Advanced NASH *n* = 14
Age (yrs), mean (SD)	50.2 (12)	50.4 (9.5)	52.3 (9.1)
**Race:**			
White, non-Hispanic (%)	100%	73%	87%
Black, non-Hispanic (%)	0%	7%	0%
Other (%)	0%	10%	3%
Not-declared (%)	0%	10%	10%
Male (%)	92%	93%	80%
Diabetes (%)	75%	71%	40%
BMI (Kg/m^2^), mean (SD)	35 (7)	33 (5)	34 (6)
**Serum Laboratory Tests, mean (SD):**			
AST (U/L)	39 (32)	39 (20)	66 (39)
ALT (U/L)	50 (30)	62 (32)	97 (42)
Albumin (g/dL)	4.4 (0.4)	4.6 (0.2)	4.6 (0.2)
Bilirubin (g/dL)	0.7 (0.4)	0.5 (0.1)	0.5 (0.3)
**Liver Histology:**			
Steatosis Grade 0/1/2/3	0/7/4/1	0/19/11/1	0/1/9/4
Inflammation Grade 0/1/2/3	3/9/0/0	0/30/1/0	0/4/10/0
Ballooning Degeneration 0/1/2	12/0/0	0/27/4	0/1/13
Fibrosis Stage 0/1/2/3/4	12/0/0/0/0	10/21/0/0/0	0/4/6/3/1
NAS Score (0–8), mean (SD)	2.4 (0.8)	3.3 (0.7)	5.7 (0.6)

**Table 2:** Metabolites that differed significantly between patients with NAFL (*n* = 12), early NASH (*n* = 31) and advanced NASH (*n* = 14).

**NASH vs. NAFL**				**Early NASH vs. NAFL**			
**Metabolite**	***p* Value**	Fold * Change	**Method**	**Metabolite**	***p* Value**	Fold * Change	**Method**
Acetylglycine	0.03	0.57	MS	Hydroxyphenylpyruvate	0.002	0.83	MS
Cysteine	0.04	0.88	MS	Inositol	0.03	0.86	MS
Alanine	0.02	0.96	NMR	Cysteine	0.04	0.87	MS
Glucose	0.04	1.16	MS	Acetylcarnitine	0.04	0.90	MS
Erythrose	0.02	1.18	MS	Phenylalanine	0.03	1.12	NMR
Tyrosine	0.01	1.18	NMR	Tyrosine	0.02	1.18	NMR
Isovaleric acid	0.02	1.25	MS	Erythrose	0.04	1.18	MS
Leucic acid	0.04	1.28	MS	Alanine	0.03	1.18	NMR
Xanthine	0.02	1.49	MS	Tryptophan	0.04	1.19	NMR
Oxypurinol	0.01	1.54	MS				
Glycochenodeoxycholate	0.04	3.13	MS				
**Advanced NASH vs. Early NASH**				**Advanced NASH vs. NAFL**			
**Metabolite**	***p* Value**	Fold * Change	**Method**	**Metabolite**	***p* Value**	Fold * Change	**Method**
Spermidine	0.005	0.49	MS	Spermidine	0.005	0.33	MS
Oxaloacetate	0.01	0.85	MS	Acetylglycine	0.01	0.48	MS
Orotate	0.0009	0.85	MS	Glucose	0.04	1.20	MS
Linoleic acid	0.01	1.32	MS	Isovaleric acid	0.04	1.30	MS
Linolenic acid	0.01	1.33	MS	Leucic acid	0.02	1.30	MS
2-hydroxyglutarate	0.01	1.33	MS	2-hydroxyisovaleric acid	0.03	1.49	MS
				Xanthine	0.04	2.08	MS
				Oxypurinol	0.04	2.17	MS
				Glycocholate	0.02	2.22	MS
				Glycochenodeoxycholate	0.01	2.38	MS

* Fold changes shown are the ratios of NASH/ NAFL; Early NASH/ NAFL; Advanced NASH/ NAFL; Advanced NASH/ Early NASH. They are ordered from the lowest ratio (i.e., most “protective” against advanced disease) to the highest ratio (i.e., most highly associated with advanced disease).

**Table 3:** Metabolites that differed significantly between patients with different levels of hepatic fibrosis (F0-1 (*n* = 43) vs. F2-4 (*n* = 14) or steatosis (grade 0-1 (*n* = 27) vs. grade 2-3 (*n* = 30)).

Fibrosis Stage 2–4 vs. Fibrosis Stage 0–1				Steatosis Grade 2–3 vs. Steatosis Grade 0–1			
Metabolite	*p* Value	Fold Change *	Method	Metabolite	*p* Value	Fold Change *	Method
Spermidine	0.0008	0.47	MS	Erythrose	0.01	1.19	MS
N-acetylglycine	0.001	0.63	MS	Mannose	0.002	1.33	NMR
Oxaloacetate	0.004	0.83	MS	Isovaleric acid	0.01	1.33	MS
Orotate	0.01	0.85	MS	Glucose	0.02/0.005	1.37/1.22	NMR/MS
Adipic acid	0.03	1.20	MS				
Sucrose	0.04	1.20	MS				
Aconitate	0.03	1.23	MS				
Azelaic acid	0.04	1.25	MS				

* The fold changes are the ratios of Fibrosis stage 2–4/Fibrosis stage 0–1; Steatosis grade 2–3/Steatosis grade 0–1. They are ordered from the lowest ratio (i.e., most “protective” against advanced disease) to the highest ratio (i.e., most highly associated with advanced disease).

## Supplementary Materials

The following are available online at https://www.mdpi.com/2218-1989/10/4/168/s1, Figure S1: Matched pathways obtained from pathway enrichment analysis and the impact of the matched pathways obtained from pathway topology analysis, Figure S2: Network view of metabolite set enrichment analysis with labeling for some of the prominent sets, Figure S3: Results of PLS-DA of metabolites derived from MS and NMR for (a) male vs female; and (b) diabetes vs non-diabetes patients, Table S1: List of metabolites quantified by mass spectrometry (MS), Table S2: List of metabolites quantified by NMR spectroscopy, Table S3: Metabolic pathways that are significantly different between patients with NAFL, early-NASH or advanced-NASH, and between different fibrosis stages or steatosis grades, Table S4: Metabolites that differed significantly between male and female patients, Table S5: Metabolites that differed significantly between diabetes and non-diabetes patients.
