# Supplementary material for: Distinguishing NASH Histological Severity Using a Multiplatform Metabolomics Approach
Source: Metabolites. 2020 Apr 24;10(4):168. doi: 10.3390/metabo10040168 (PMC7240949; doi:10.3390/metabo10040168)
Supplement: Supplementary file 1 [file metabolites-10-00168-s001.pdf]

Supplementary Material

Distinguishing NASH Histological Severity Using a Multiplatform Metabolomics Approach

George N. Ioannou[1,2,3*] G. A. Nagana Gowda,[4] Danijel Djukovic,[4] and Daniel Raftery[4,5,6*]

1 Division of Gastroenterology, Veterans Affairs Puget Sound Healthcare System and University of Washington, Seattle WA; GeorgeI@medicine.washington.edu (G.N.I)

2 Department of Medicine, Veterans Affairs Puget Sound Healthcare System and University of Washington, Seattle WA.

3. Research and Development, Veterans Affairs Puget Sound Healthcare System, Seattle WA.

4 Northwest Metabolomics Research Center, Anesthesiology and Pain Medicine, University of Washington, Seattle, WA 98109; ngowda@uw.edu (G.A.N.G); djukovic@uw.edu (D.D.); raftery@uw.edu (D.R.).

5. Department of Chemistry, University of Washington, Seattle, WA 98195.

6. Fred Hutchinson Cancer Research Center, Seattle, WA 98109.

* Correspondence:

G.N.I.: GeorgeI@medicine.washington.edu; Tel.: +1-206-277-3136;

D.R.: draftery@uw.edu; Tel.:+1- 206-543-9709

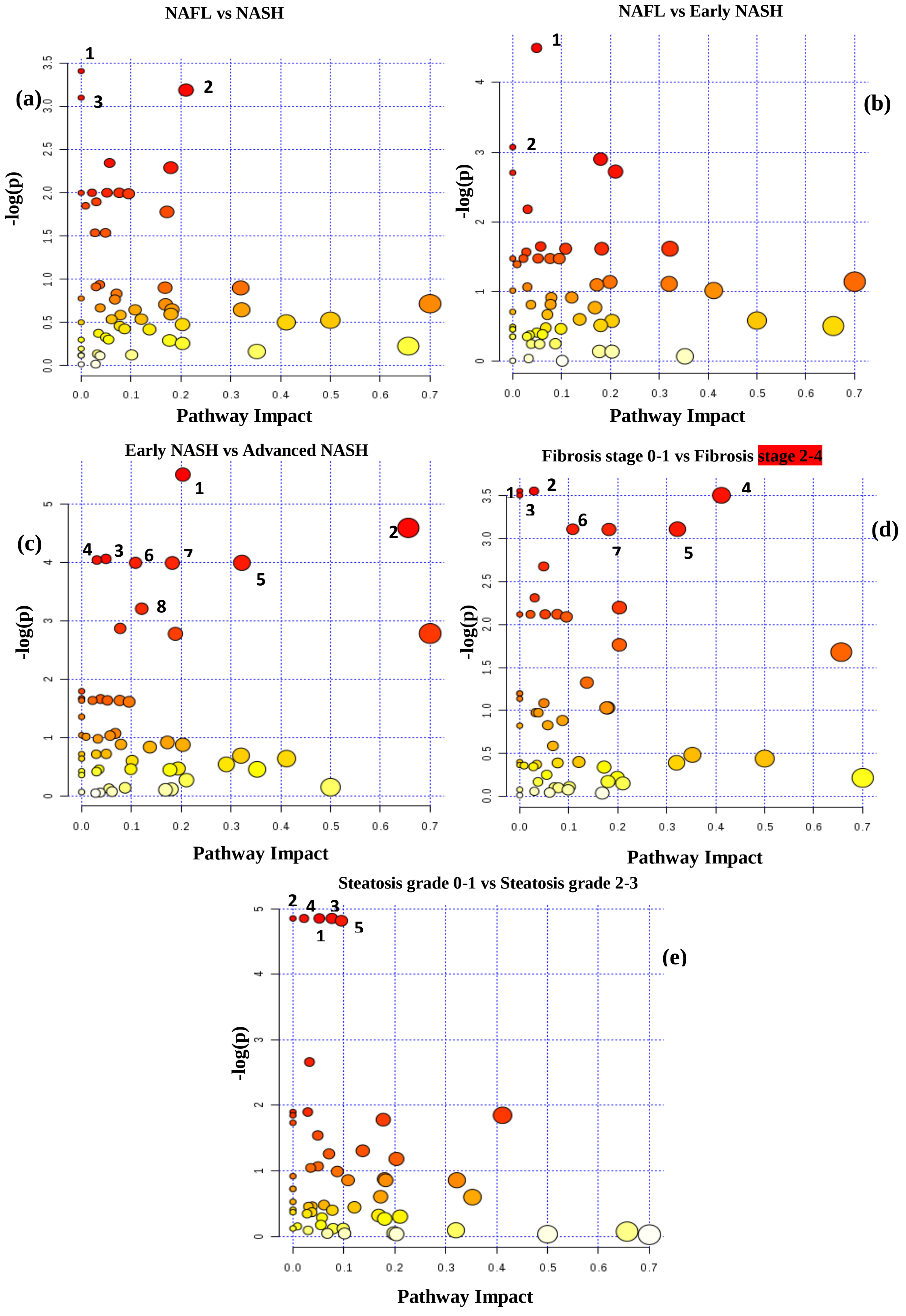

NAFL vs NASH
(a)
-log(p)
Pathway Impact
NAFL vs Early NASH
(b)
-log(p)
Pathway Impact
Early NASH vs Advanced NASH
(c)
-log(p)
Pathway Impact
Fibrosis stage 0-1 vs Fibrosis stage 2-4
(d)
-log(p)
Pathway Impact
Steatosis grade 0-1 vs Steatosis grade 2-3
(e)
-log(p)
Pathway Impact

Figure S1: Matched pathways obtained from pathway enrichment analysis and the impact of the matched pathways obtained from pathway topology analysis. Each pathway is shown as a circle. The color of the pathway indicates the significance (p-value) of the pathway with red being the most significant ($p<0.05$) and the size of the circle indicates the impact of the pathway. The results shown here were obtained from the analysis based on 106 mass spectrometry (MS) derived metabolites, which showed a total of 56 matched pathways to distinguish (see also Table S3): (a) NAFL vs. NASH; 3 pathways (labeled 1-3) were significantly different ($p<0.05$): Fatty acid biosynthesis (1); Tryptophan metabolism (2); Nitrogen metabolism (3); (b) NAFL vs. early NASH; 2 pathways (labeled 1, 2) were significantly different ($p<0.05$): Ubiquinone and other terpenoid-quinone biosynthesis (1); Fatty acid biosynthesis (2); (c) Early NASH vs. advanced NASH; 8 pathways (labeled 1-8) were significantly different ($p<0.05$): alpha-Linolenic acid metabolism (1); Linoleic acid metabolism (2); Ubiquinone and other terpenoid-quinone biosynthesis (3); Caffeine metabolism (4); Citrate cycle (TCA cycle) (5); Alanine, aspartate and glutamate metabolism (6); Glyoxylate and dicarboxylate metabolism (7); Pyrimidine metabolism (8); (d) Fibrosis stage 0,1 vs. Fibrosis stage 2,3,4; 7 pathways (labeled 1-7) were significantly different ($p<0.05$): Porphyrin and chlorophyll metabolism (1); Valine, leucine and isoleucine biosynthesis (2); Aminoacyl-tRNA biosynthesis (3); Glycine, serine and threonine metabolism (4); TCA cycle (5); Alanine, aspartate and glutamate metabolism (6); Glyoxylate and dicarboxylate metabolism (7); and (e) Steatosis grade 0,1 vs. Steatosis grade 2,3; 5 pathways (labeled 1-5) were significantly different ($p<0.05$): Galactose metabolism (1); Amino sugar and nucleotide sugar metabolism (2); Starch and sucrose metabolism (3); Pentose phosphate pathway (4); Glycolysis and gluconeogenesis (5).

(a) Advanced NASH vs NAFL

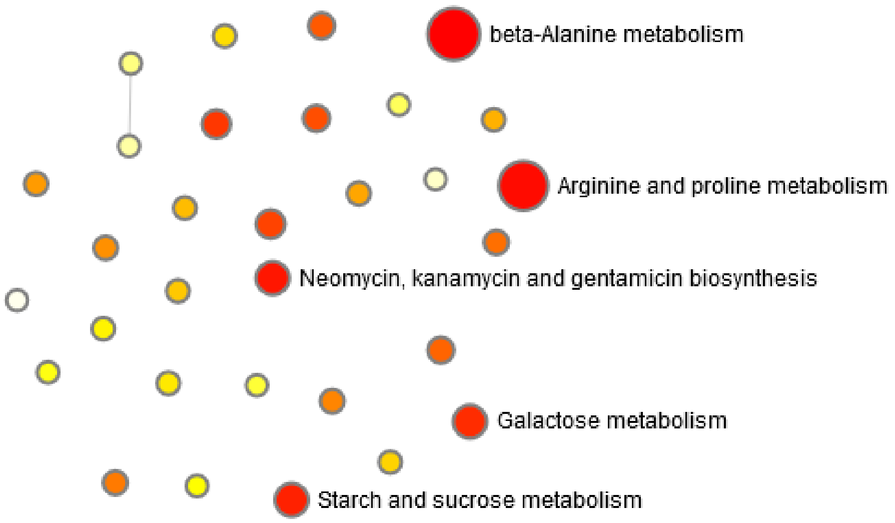

(b) Early NASH vs Advanced NASH

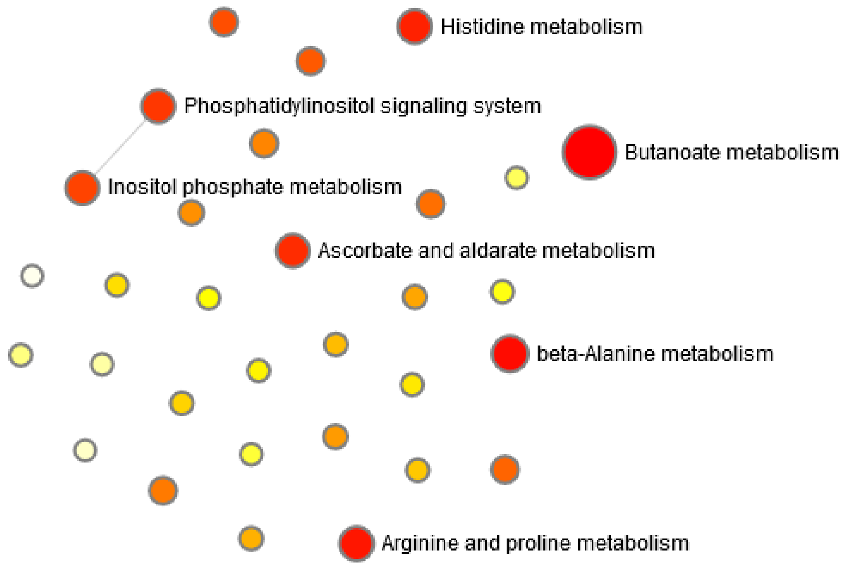

Figure S2: Network view of metabolite set enrichment analysis with labeling for some of the prominent sets. Metabolite data were derived from MS. In the figure, each node represents a metabolite set. The color and size indicate the p value and the relative number of hits to the query, respectively. Metabolite sets that are connected by an edge indicate that the number of their shared metabolites is >25% of the total number of their combined metabolites sets. Between advanced NASH and NAFL groups (Fig. S2a), 31 sets were identified of which two sets exhibited high significance. These two sets correspond to (1) beta-alanine metabolism and (2) arginine and proline metabolism; the p value adjusted using false discovery rate for both sets was 0.02. Between early NASH and advanced NASH groups (Fig. S2b), 31 sets were identified of which one set exhibited high significance. This set corresponds to butanoate metabolism and the p value adjusted using the false discovery rate was 0.05.

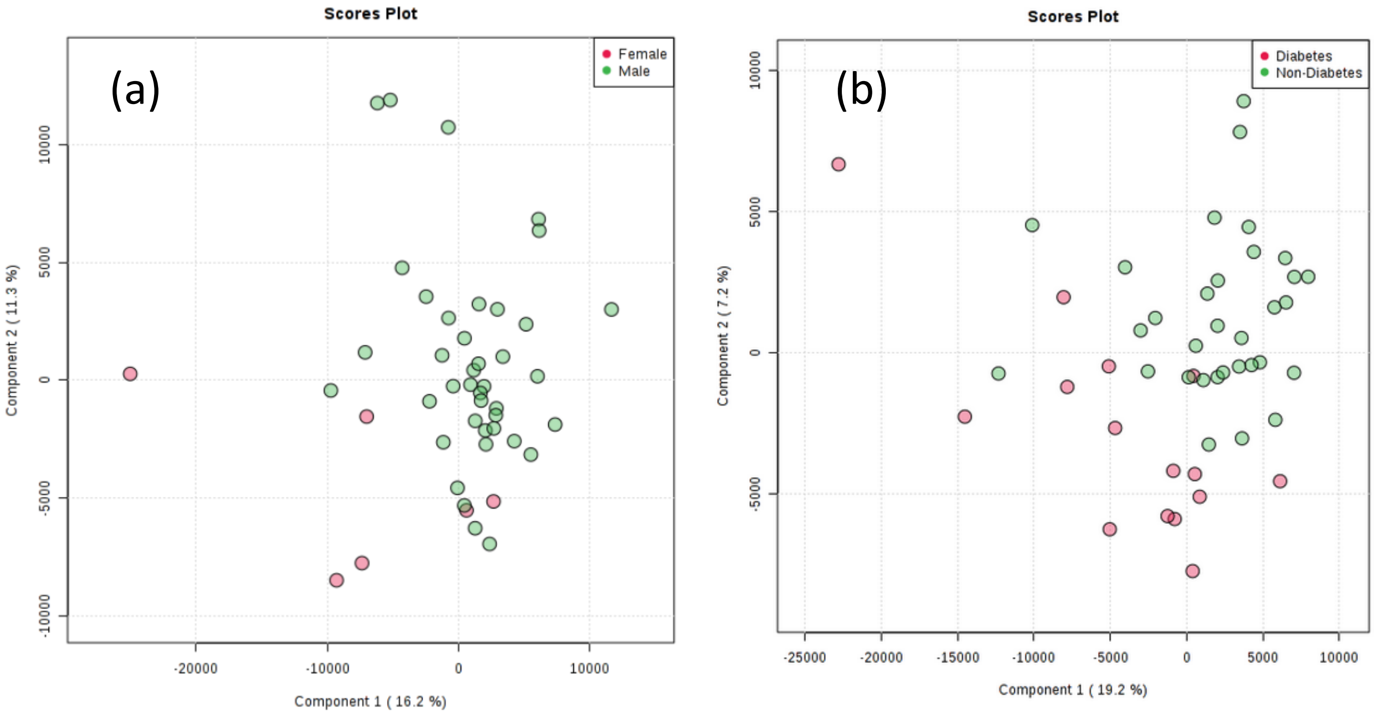

Figure S3: Results of PLS-DA of metabolites derived from MS and NMR for (a) male vs female; and (b) diabetes vs non-diabetes patients.

Table S1: List of metabolites quantified by MS. The first number in the parenthesis indicates the m/z value for precursor ion and the second number indicates the value for product ion.

1	1/3-Methylhistidine (170.0 / 96.0)	54	Guanidinoacetate (116.0 / 74.0 (2))
2	12-HETE (319.2 / 179.0)	55	Histamine (112.0 / 95.0)
3	13-HODE (295.2 / 195.0)	56	Homocysteine (136.0 / 90.0)
4	1-Methyladenosine (282.0 / 150.1)	57	Homoserine (120.1 / 74.0)
5	1-Methylguanosine (298.0 / 166.1)	58	Hydroxyproline/Aminolevulinate (132.1 / 86.2)
6	1-Methylhistamine (126.0 / 96.0)	59	Hypoxanthine (135.0 / 92.0)
7	2-Aminoadipate (160.1 / 116.0)	60	Hyppuric Acid (178.0 / 134.0)
8	2-Hydroxyglutarate (147.0 / 129.0)	61	Inosine (269.0 / 137.1)
9	2-Hydroxyisovaleric Acid (117.0 / 71.0)	62	Inositol (179.0 / 87.0)
10	3-Hydroxybutyric acid (103.0 / 59.0)	63	Isovaleric Acid (101.0 / 83.0)
11	4-Hydroxybutyrate (105.0 / 77.0)	64	Kynurenate (188.0 / 143.8)
12	4-Pyridoxic acid (182.1 / 138.0)	65	Lactate (89.0 / 43.0)
13	5-Hydroxytryptophan (221.0 / 133.2)	66	Lactose (341.0 / 59.0)
14	Acetoacetate (101.0 / 57.0)	67	Linoleic Acid (277.1 / 259.0)
15	Acetylcarnitine (204.1 / 85.0)	68	Linolenic Acid (279.1 / 261.0)
16	Acetylcholine (146.1 / 87.0)	69	L-Kynurenine (209.1 / 94.0)
17	Aconitate (173.0 / 85.0)	70	Malonic Acid (103.0 / 41.0)
18	Adenosine (268.1 / 136.1)	71	Margaric Acid (269.1 / 251.3)
19	Adenylosuccinate (462.1 / 79.0)	72	Mevalonate (147.1 / 59.0)
20	Adipic Acid (144.9 / 83.0)	73	Myristic Acid (227.1 / 209.0)
21	Agmanite (131.0 / 72.0)	74	N2,N2-Dimethylguanosine (312.0 / 180.1)
22	Allantoin (157.0 / 114.0)	75	N-Acetylglycine (116.0 / 74.0)
23	Alpha-Ketoglutaric Acid (145.0 / 101.0)	76	Niacinamide (123.0 / 80.0)
24	Aminoisobutyrate (104.1 / 86.0)	77	Nicotinate (Niacin) (122.0 / 78.0)
25	Anthranilate (136.0 / 118.0)	78	OH-Phenylpyruvate (179.0 / 89.0 (4))
26	Azelaic Acid (187.0 / 125.0)	79	Ornithine (133.1 / 70.0)
27	Benzoic acid (121.0 / 77.0 (2))	80	Orotate (155.0 / 111.0)
28	Biotin (243.1 / 200.0)	81	Oxalacetate (131.0 / 113.0)
29	Cadaverine (103.0 / 86.0)	82	Oxalic Acid (89.0 / 61.0)
30	Citraconic Acid (129.0 / 85.0)	83	Oxypurinol (151.0 / 42.0)
31	Citrulline (174.0 / 131.0)	84	Pentothenate (218.1 / 88.0)
32	CMP (322.0 / 97.0)	85	Pipecolate (130.0 / 84.0)
33	Cystamine (153.0 / 108.0)	86	Propionate (73.0 / 55.0)
34	Cysteine (122.0 / 59.0)	87	Pyroglutamic Acid (130.0 / 83.4)
35	Cystine (241.1 / 120.0)	88	Pyruvate (87.0 / 43.0)
36	Cytidine (244.0 / 112.1)	89	Quinolinate (166.0 / 78.0)
37	Deoxycarnitine (147.1 / 87.0)	90	Sarcosine (89.9 / 44.0)

38 D-GA3P (168.9 / 97.0)
39 D-Leucic Acid (131.0 / 85.0)
40 Epinephrine/Normetanephrine (184.1 / 166.2)
41 Erythrose (119.0 / 71.0)
42 F16BP/F26BP/G16BP (339.0 / 97.0)
43 G1P/G6P/F6P/F1P (259.0 / 97.0)
44 Gama-aminobutyrate (102.1 / 56.0)
45 Glucoronate (193.0 / 73.0)
46 Glucose (179.0 / 89.0)
47 Glutaric Acid (131.0 / 87.0)
48 Glyceraldehyde (89.0 / 59.0)
49 Glycerate (105.0 / 75.0)
50 Glycerol-3-P (171.0 / 79.0)
51 Glycine (76.0 / 30.1)
52 Glycochenodeoxycholate (448.3 / 74.0)
53 Glycocholate (464.3 / 74.0)
91 Shikimic Acid (173.0 / 93.0)
92 Sorbitol (183.0 / 91.0)
93 Spermidine (146.1 / 72.0)
94 Succinate (117.0 / 73.0)
95 Sucrose (341.0 / 59.0 (2))
96 Taurine (126.0 / 108.0)
97 Trimethylamine (TMA) (60.0 / 44.0)
98 Trimethylamine-N-oxide (TMAO) (76.1 / 58.0)
99 Tryptamine (161.1 / 144.0)
100 Tryptophan (205.1 / 146.0)
101 Tyramine (138.1 / 77.0)
102 Urate (167.0 / 124.0)
103 Uridine (245.0 / 113.1)
104 Xanthine (151.0 / 108.0)
105 Xanthosine (283.1 / 151.0)
106 Xanthurenate (204.0 / 160.0)

Abbreviations:

HODE: Hydroxyoctadecadienoic acid

HETE: Hydroxyeicosatetraenoic acid

CMP: Cytidine monophosphate

GA3P: Glyceraldehyde 3-phosphate

F16BP: Fructose 1,6-bisphosphate

F26BP: Fructose: 2,6-bisphosphate

G16BP: Glucose 1,6-bisphosphate

G1P: Glucose 1-bisphosphate

G6P: Glucose 6-bisphosphate

F6P: Fructose 6-bisphosphate

F1P: F6P Fructose 1-bisphosphate

Table S2: List of metabolites quantified by NMR spectroscopy.

1	1,2-Propanediol	19	Citrate	37	Lysine
2	2-Aminobutyric acid	20	Creatine	38	Mannose
3	2-Hydroxybutyrate	21	Creatinine	39	Methionine
4	2-Hydroxyisovaleric acid	22	Dimethylglycine	40	Myoinositol
5	2-Oxoisocaproic acid	23	Formic acid	41	N-Acetylglycine
6	2-Oxoisovaleric acid	24	Fumaric acid	42	Ornithine
7	3-Hydroxybutyric acid	25	Glucose	43	Phenylalanine
8	3-methyl-2-oxovaleric acid	26	Glutamic acid	44	Proline
9	3-Methylhistidine	27	Glutamine	45	Pyroglutamic acid
10	Acetate	28	Glycerol	46	Sarcosine
11	Acetylcarnitine	29	Glycine	47	Sucrose
12	Alanine	30	Histidine	48	Threonine
13	Arginine	31	Hypoxanthine	49	Tryptophan
14	Asparagine	32	Isobutyric acid	50	Tyrosine
15	Aspartate	33	Isoleucine	51	Uridine
16	Betaine	34	Isovaleric acid	52	Valine
17	Carnitine	35	Lactate		
18	Choline	36	Leucine		

Table S3: Metabolic pathways that are significantly different between patients with NAFL, early-NASH or advanced-NASH, and between different fibrosis stages or steatosis grades.

NAFL vs NASH

Pathway	P value	Method
Fatty acid biosynthesis	0.03	MS
Tryptophan metabolism	0.04	MS
Nitrogen metabolism	0.04	MS
Cysteine and methionine metabolism	0.04	NMR
Taurine and hypotaurine metabolism	0.05	NMR
Selenoamino acid metabolism	0.05	NMR

NAFL vs Early NASH

Pathway	P value	Method
Ubiquinone and other terpenoid-quinone biosynthesis	0.01	MS
Fatty acid biosynthesis	0.04	MS

Early NASH vs Advanced NASH

Pathway	P value	Method
Alpha-Linolenic acid metabolism	0.004	MS
Linoleic acid metabolism	0.01	MS
Ubiquinone and other terpenoid-quinone biosynthesis	0.01	MS
Caffeine metabolism	0.01	MS
TCA cycle	0.01	MS
Alanine, aspartate and glutamate metabolism	0.01	MS
Glyoxylate and dicarboxylate metabolism	0.01	MS
Pyrimidine metabolism	0.04	MS

Hepatic fibrosis stage 0-1 vs Fibrosis stage 2-4

Pathway	P value	Method
Porphyrin and chlorophyll metabolism	0.03	MS
Valine, leucine and isoleucine biosynthesis	0.03	MS
Aminoacyl-tRNA biosynthesis	0.03	MS
Glycine, serine and threonine metabolism	0.03	MS
TCA cycle	0.04	MS
Alanine, aspartate and glutamate metabolism	0.04	MS
Glyoxylate and dicarboxylate metabolism	0.04	MS

Steatosis grade 0-1 vs Steatosis grade 2-3

Pathway	P value	Method
Galactose metabolism	0.007	MS
Amino sugar and nucleotide sugar metabolism	0.007/0.02	MS/NMR
Starch and sucrose metabolism	0.007/0.02	MS/NMR
Pentose phosphate pathway	0.007/0.02	MS/NMR
Glycolysis and gluconeogenesis	0.008/0.02	MS/NMR
Fructose and mannose metabolism	0.003	NMR

Table S4: Metabolites that differed significantly between male and female patients.

**Metabolite**	**P Value**	**Fold change***	**Method**
Glycine	0.004	0.70	MS
Glutamic acid	0.010	1.27	NMR
Leucine	0.009	1.23	NMR
Isoleucine	0.004	1.41	NMR
Lysine	0.01	1.27	NMR
3-methyl-2-oxovaleric acid	0.02	1.37	NMR
2-Oxoisocaproic acid	0.03	1.34	NMR
Lactate	0.02	1.41	NMR
Formic acid	0.009	1.37	NMR
Choline	0.03	1.46	NMR
Hypoxanthine	0.01	2.08	NMR
Proline	0.003	1.40	NMR
Glycerol	0.04	0.63	NMR
Creatinine	0.03	1.28	NMR
2-hydroxybutyrate	0.03	1.83	NMR
Trimethylamine-N-oxide	0.02	0.50	MS
13-HODE*	0.004	0.68	MS

* The fold changes are the ratios of male/female patients.
HODE: Hydroxyoctadecadienoic acid

Table S5: Metabolites that differed significantly between diabetes and non-diabetes patients.

**Metabolites**	**P Value**	**Fold change***	**Method**
Glycine	0.05	0.8	NMR
Hydroxyproline/Aminolevulinate	0.004	1.4	MS
Propionate	0.02	1.4	MS
Isovaleric Acid	0.05	1.3	MS
Guanidinoacetate	0.009	0.8	MS
Mevalonate	0.004	1.5	MS
Oxypurinol	0.04	1.5	MS
Xanthine	0.05	1.5	MS
2-Aminoadipate	0.03	1.3	MS
Sucrose	0.04	1.1	MS

* The fold changes are the ratios of diabetes/non-diabetes patients.
